# Public Health Burden of Secondhand Smoking: Case Reports of Lung Cancer and a Literature Review

**DOI:** 10.3390/ijerph192013152

**Published:** 2022-10-13

**Authors:** Ladislav Štěpánek, Jarmila Ševčíková, Dagmar Horáková, Mihir Sanjay Patel, Radka Durďáková

**Affiliations:** Department of Public Health, Faculty of Medicine and Dentistry, Palacký University Olomouc, Hněvotínská 3, 779 00 Olomouc, Czech Republic

**Keywords:** secondhand smoke, mainstream smoke, sidestream smoke, lung cancer, childhood, smoking status

## Abstract

Secondhand smoke (SHS), composed of mainstream and sidestream smoke, is a known human carcinogen. It contains a variety of harmful substances at even higher concentrations than mainstream smoke itself, which is inhaled during firsthand smoking. Exposure to SHS, affecting more than a third of the worldwide population, increases the likelihood of lung cancer by roughly 30%, with specific contributions depending on the histological type of cancer. This study aimed to present the harmful potential of SHS through case reports and describe the burden of SHS via a literature review. From a collection of lung cancer case reports occurring in never smokers from the Olomouc district over the last 10 years, 2 cases with no risk factors for lung cancer except for significant exposure to SHS were identified. Both cases were of young women who lived in households where their parents smoked during childhood. They suffered from rarer histological types of lung cancer in which the association with SHS has not yet been analyzed. As the literature confirms, SHS has the most adverse effects in individuals exposed during childhood. It is necessary to both take measures to reduce the prevalence of SHS, especially among children in households and pay due attention to the smoking history of patients, including current and previous exposure to SHS, when obtaining anamnestic data. Furthermore, the effect of SHS on rarer histological types of lung cancer should be studied.

## 1. Introduction

Lung cancer is the leading cause of global cancer incidence and mortality, accounting annually for an estimated 2 million diagnoses and 1.8 million deaths. The relationship between tobacco smoking and lung cancer development has already been reliably proven [1]. A meta-analysis of 99 cohort studies found that when compared with non-smoking, current firsthand smoking was associated with an age-adjusted relative risk (RR) of lung cancer of 7.48 (95% confidence interval [CI] 5.29–10.60) in women and 8.78 (95% CI 6.13–12.57) in men. The risk of lung cancer increased according to the number of cigarettes smoked per day in both sexes. In women, the RRs were 5.30 (95% CI 3.52–7.97), 10.67 (95% CI 7.43–15.33) and 17.09 (95% CI 12.11–24.11) across subgroups of <10, 10 to 20 and >20 cigarettes per day versus non-smoking. Corresponding RRs in men were 4.97 (95% CI 2.74–9.03), 8.93 (95% CI 4.90–16.28) and 14.61 (95% CI 8.33–25.59) [2]. The association between smoking and lung cancer differs greatly by histological type of cancer. Specifically, in smokers with an average daily dose of >30 cigarettes, a pooled analysis including 13,169 cases and 16,010 controls from Europe and Canada quantified the odds ratio (OR) of 103.5 (95% CI 74.8–143.2) for squamous cell carcinoma, 111.3 (95% CI 69.8–177.5) for small cell lung cancer, and 21.9 (95% CI 16.6–29.0) for adenocarcinoma among men. In women, the corresponding ORs were 62.7 (95% CI 31.5–124.6), 108.6 (95% CI 50.7–232.8), and 16.8 (95% CI 9.2–30.6), respectively [1].

Exposure to cigarette smoke can take many forms in terms of both quantity and quality [1,3]. In addition to firsthand smoke being proven as a risk factor, secondhand smoke (SHS) has also been classified as a “Group 1” carcinogen (known human carcinogen) by the International Agency for Research on Cancer and has been shown to have several adverse health effects on adults and children [4].

In 2017, 0.36% of overall disability-adjusted life years and 0.46% of all deaths were attributable to household SHS exposure in the 28 European Union countries [5]. As with firsthand smoking, a link between SHS and lung cancer has been demonstrated [6]. Besides cancer, the respiratory tract exposed to SHS becomes more susceptible to other disorders [7]. Exposure to SHS, however, is also reflected in an increased risk of cardiovascular diseases [8] and malignant processes in other body systems [9,10,11,12], although the exact impact of SHS on all body systems remains unclear. Recent smoke-free policies have reduced the burden of SHS, mostly in public places, but a significant portion of the global population remains in health-threatening exposure to SHS [5,13].

This study aimed to present the harmful potential of SHS through case reports of patients suffering from lung cancer in which SHS was the only risk factor and to describe the burden of SHS via a brief literature review.

## 2. Methods

In April 2022, general practitioners (GP) of the Olomouc district (± 230,000 habitants) were asked to provide information on all cases of lung cancer that they had noted in never smokers over the last 10 years. Via e-mail, GPs were specifically asked about any risk factors for lung cancer in the provided cases (N = 27), namely, family occurrence as well as environmental and occupational factors. In two cases, only exposure to SHS was detected. These cases were then analyzed in detail. We conducted a structured, non-exhaustive search of the literature. The search terms for this study included “secondhand smoke” (including hyphenated variations on spelling), as well as “prevalence”, ”composition”, “cancer”, ”cardiovascular”, and “measures”. Electronic databases, including MEDLINE/PubMed, Scopus, and Web of Science, were searched to identify the most relevant studies with a preference for the most recent papers written in English.

## 3. Case Reports

### 3.1. Case 1

In May 2021, a GP examined a 24-year-old female patient for painful resistance on the left side of her neck, which the patient noticed for the first time a week ago. The diameter of the hard resistance, located behind the sternocleidomastoid muscle, was 20 millimetres. The patient’s physical examination showed no abnormalities, as did a chest skiagram, blood count with differential leukocyte count, thyroid hormones, serology of Epstein-Barr virus, cytomegalovirus, and toxoplasmosis. Only C-reactive protein (CRP) was elevated to 63.2 mg/L with a subsequent decrease to 7.9 mg/L in 4 days. Ultrasound showed a packet of enlarged oval hypoechoic lymph nodes of an inhomogeneous structure near the lower edge of the left sternocleidomastoid muscle (the largest node with a diameter of 18 millimetres). The patient was medicated with amoxicillin/acidum clavulanic.

In July 2021, the patient reported a reduction in the cervical lymphadenopathy (greatest diameter reported by GP was 15 mm), but with new onset of heartburn and a mild cough without expectoration; the patient otherwise presented in a stable condition. Her blood count showed no abnormalities, and her CRP was 18.2 mg/L. A repeated ultrasound examination verified the original finding, with the total size of the lymphadenopathy presenting at 36 × 15 × 27 mm. In September 2021, the patient underwent extirpation of the nodes with the subsequent histological finding of a branchiogenic cyst with flattened epithelium without atypia and almost a complete consumption of the nodes by metastasis of mucinous adenocarcinoma. Positron emission tomography and computed tomography (PET CT) showed a radiopharmaceutical accumulation in the left lower lobe of the lung measuring 12 × 11 × 13 mm extending to the pleura, other accumulations in both lungs in numerous small nodules up to 4 mm, in left supraclavicular lymph nodes merging into packets, in lymph nodes of all mediastinal compartments and both lung hila, in the right lobe of the liver, and the upper pole of the spleen. Magnetic resonance imaging of the brain revealed suspicious foci in the right parietal and left cerebellar hemispheres. The diagnosis was concluded as left-sided bronchogenic mucinous adenocarcinoma with dissemination. In October 2021, therapy with alectinib (anaplastic lymphoma kinase inhibitor) was started with a subsequent finding of significant regression of the described foci and no evidence of new ones.

The patient, a never smoker, had shared a household with her father, who smoked 15 cigarettes a day, all her life. The father smoked in the interior of their family house. The patient had never been significantly exposed to cigarette smoke elsewhere. Personal history included prematurity (delivery at 30 weeks with subsequent neonatal jaundice) with reaching normal psychomotor development, puncture of an anechoic cyst under the jaw on the right without histological evidence of abnormal cells in 2019, and the presence of a stable small nodule of the right lobe of the thyroid gland. Her family history did not include oncological diseases. Significant exposure to other lung cancer risk factors (environmental, occupational) was not detected.

### 3.2. Case 2

In April 2015, a 34-year-old female patient developed a week-lasting cough, evening wheezing, diarrhoea, and fever. Physical and basic laboratory examinations did not reveal any abnormalities except for a concentration of CRP above the normal range (12.2 mg/L). During the initial therapy (inhaled salbutamol, peroral levocetirizine and nifuroxazide), the diarrhoea subsided within a week, but the cough persisted despite subsequent strengthening of inhalation therapy with budesonide. In the auscultatory findings of the lungs, isolated murmurs appeared bilaterally. Chest skiagram (initial and repeated after 3 weeks) showed a soft round shadow measuring 12 mm in the right middle lung field.

A sharply demarcated mass in the right middle lobe and a triangular-shaped consolidation of the lung parenchyma in the left lower lobe of the nature of post-inflammatory changes were visible on the CT. The PET CT performed in August 2015 demonstrated a radiopharmaceutical accumulation in a spherical deposit in the right middle lobe measuring 10 mm. Therefore, a video-assisted thoracoscopic lobectomy was performed in September 2015. The histological examination concluded the diagnosis as a poorly differentiated carcinoma with sarcomatoid features without the presence of metastases in the mediastinal nodes. Repeated PET CT scans did not detect disease recurrence.

The patient had never smoked, but until the age of 10, she had lived in a household with a father smoking 20 cigarettes a day including in the interior of their family house. She had another non-negligible exposure to SHS between 2005 and 2015 at her workplace. The patient’s personal history only included the extirpation of a benign breast fibroadenoma. Her family history did not include oncological diseases. Significant exposure to other lung cancer risk factors (environmental, occupational) was not detected.

## 4. Discussion and Literature Review

Both patients represent cases of rarer histological types of lung cancer. Sarcomatoid carcinoma is usually diagnosed in heavy smokers, but the link between mucinous adenocarcinoma and smoking is not clear [14,15]. However, no risk factor for lung cancer was detected in either patient except for their significant SHS exposure. To the best of our knowledge, the association of these histological types with SHS has not been analyzed in the available literature.

Exposure to SHS is a widespread public health burden. More than a third of the global population are passive smokers and are regularly exposed to the dangerous effects of tobacco smoke [16]. Based on surveys conducted in 142 countries between 1 January 2010 and 31 December 2018, the global prevalence of SHS exposure in any place was 62.9% on 1 or more days, 51.0% on 3 or more days, 40.1% on 5 or more days, and 32.5% daily during the past 7 days. The prevalence of SHS exposure at home was 33.1% on 1 or more days, 20.1% on 3 or more days, 14.9% on 5 or more days, and 12.3% daily during the past 7 days; in public places, the corresponding prevalence rates were 57.6%, 43.4%, 30.3%, and 23.5%, respectively [17]. Women are more likely than men to be exposed to SHS [18]. The highest rates of significant SHS exposure among never smokers were reported in middle-income followed by low-income and high-income countries [19]. Specifically for children, a study across 192 countries showed that 40% of children were exposed to SHS and 36% were exposed to SHS in utero, especially in countries with high smoking prevalence and among rural populations [16,20]. Low socioeconomic status and less education were associated with a higher prevalence of SHS exposure [21,22]. Moreover, SHS was positively correlated with undesirable social phenomena such as household food insecurity [23].

Tobacco is estimated to kill more than 8 million people each year. Around 7 million of those deaths are the result of direct tobacco use while around 1.2 million are the result of non-smokers being exposed to SHS [24]. According to data from 2004, 73% of deaths from SHS exposure occurred in adults (47% in women, 26% in men) and 28% in children [25]. However, there is a substantial positive trend, as the proportion of children younger than 5 years dying from SHS exposure dropped from 29% of total deaths of SHS exposure in 1990 to 6% in 2016 [26].

**Figure 1:**
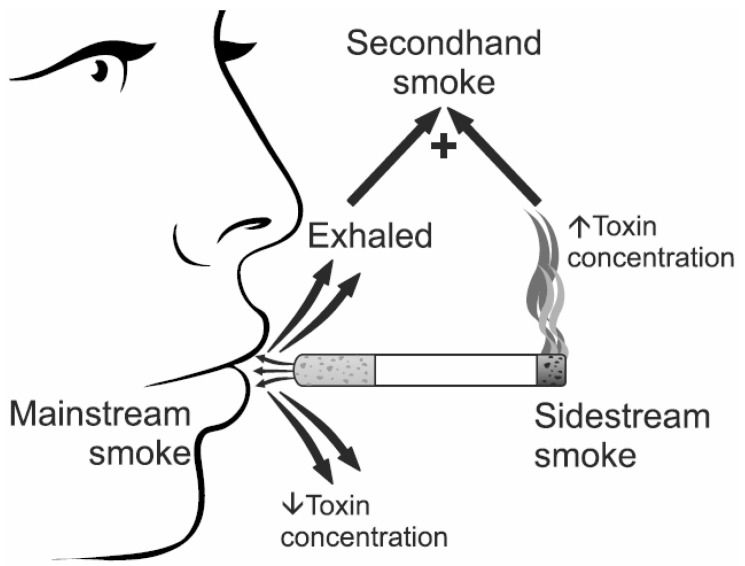
Composition of second-hand smoke. Sidestream smoke, containing toxins in higher concentrations compared to mainstream smoke, forms more than 80% of secondhand smoke.

**Figure 2:**
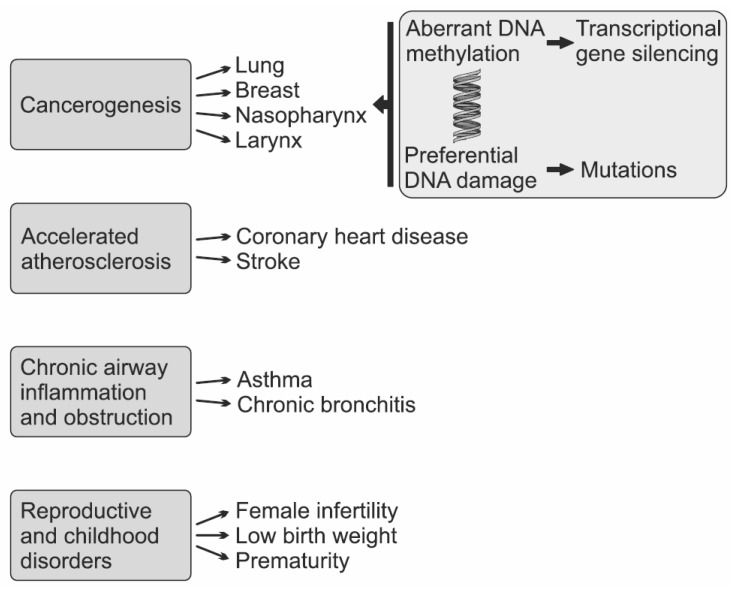
Proven health effects of secondhand smoke and the mechanism of its action on genes.

SHS is a combination of mainstream smoke breathed out by smokers and sidestream smoke from the burning end of a cigarette (Figure 1) [27]. Chemically, 80–85% of SHS is composed of sidestream smoke, and the remainder is exhaled in mainstream smoke [18,28]. Although the compositions of sidestream and mainstream smoke are qualitatively similar, there are substantial quantitative differences in composition between them as chemicals emitted from tobacco change with temperature, oxygen concentration, pH, and the extent of combustion [28]. In comparison with mainstream smoke, sidestream smoke burns at a lower temperature, causing incomplete combustion of tobacco. SHS contains more than 7000 chemicals, of which hundreds are toxic and at least 70 have been proven to cause cancer [27]. Most compounds from cigarettes are emitted in sidestream smoke at much higher concentrations than in mainstream smoke. Generally, mean levels of alcohols and phenols in sidestream smoke are higher than those reported for mainstream smoke [29]. The amount of total polycyclic aromatic hydrocarbons in sidestream smoke is about tenfold higher compared with mainstream smoke [28]. The ratio of the mass of benzene emitted into sidestream smoke compared to that emitted into mainstream smoke is approximately 10, while the corresponding ratio for 4-aminobiphenyl is 30, and that for nicotine is approximately 2. Sidestream smoke is also known to contain benzopyrene, one of the typical carcinogens, and N-nitroso-dimethyl alanine at concentrations that are 4.5 times and a phenomenal 100 times greater than that in mainstream smoke, respectively [18]. Sidestream emissions of chemicals are quite similar among a wide range of cigarette brands and styles, including regular, unfiltered, filtered, and “low tar, low nicotine” cigarettes [28]. However, except for tobacco, other components of a commercial cigarette (filter, paper, or additives) may influence the chemical content of SHS [29]. Animal experiments by Philip Morris laboratories demonstrated that sidestream smoke was three to four times more toxic than mainstream smoke [28]. The application of a concentrated substance obtained from sidestream smoke to the skin of mice was shown to more readily cause skin cancer than a concentrated substance obtained from mainstream smoke [18]. Two distinct pathways of genotoxic and epigenetic effects of SHS have been hypothesized, which might contribute to human lung cancer development, including the formation of persistent DNA damage at key cancer-related genes, and aberrant DNA methylation (global hypomethylation or locus-specific CpG island hypermethylation), respectively (Figure 2) [30].

Considering these findings, it is reasonable to trace the relationship between SHS exposure and its adverse effects on health. Since the publication of initial reports in the 1980s, SHS has been shown to increase the risk of lung cancer and gradually other types of cancer. Analytical studies across continents have overwhelmingly demonstrated a causal relationship between SHS and lung cancer and the value of RR is fairly stable over time. A recent meta-analysis of the adjusted data from 40 (mostly case-control) studies showed a hazard ratio of 1.22 (95% CI 1.13–1.33) for overall cancer in secondhand smokers compared with non-smokers. Further subgroup analyses exploring the association of SHS with different types of cancer yielded OR of 1.25 (95% CI 1.03–1.51) for 12 studies on lung cancer and OR of 1.24 (95% CI 1.10–1.39) for 15 studies on breast cancer. The associations were stronger in the case of women. No other cancer types were statistically significantly related to SHS in the study [18]. Like the effect of firsthand smoking on the development of various histological types of lung cancer, it is also possible to observe different effects of exposure to SHS on the histological types. In a pooled analysis of 18 case-control studies, the OR comparing those ever exposed to SHS with those never exposed was 1.31 (95% CI 1.17–1.45) for all histological types combined, 1.26 (95% CI 1.10–1.44) for adenocarcinoma, 1.41 (95% CI 0.99–1.99) for squamous cell carcinoma, 1.48 (95% CI 0.89–2.45) for large cell lung cancer, and 3.09 (95% CI 1.62–5.89) for small cell lung cancer. The estimated association with SHS exposure was greater for small cell lung cancer than for non-small cell lung cancers (OR 2.11, 95% CI 1.11–4.04) [31]. A more dominant effect of the exposure on the development of small cell carcinoma among the other histological types was described in the introduction for firsthand smoking as well. Our patients represented rare histological types, in which the relationship to SHS has not been analyzed according to the available literature. The differences in the strength of associations by histological types are thought to be related to tumour location too. Small cell lung cancer mainly occurs in the large central bronchi, where a high deposition of cigarette smoke particles is expected due to aerodynamic diameters [31]. Our presented cases had peripherally located lung tumours, where we should therefore assume a weaker accumulation of harmful smoke components.

Figure 2 summarizes the health effects of SHS which have been proven by scientific evidence. The role of SHS in the risk of cancer in non-smokers has been investigated mainly for lung cancer, with considerably less data on other cancer sites. Except for the aforementioned breast cancer, exposure to passive smoking was independently associated with an increased risk of nasopharyngeal, laryngeal, and cervical cancer [32,33,34,35]. Moreover, SHS also predisposes to a higher cardiovascular (CV) morbidity. For instance, 55 eligible studies were included in a meta-analysis to investigate the association between SHS exposure and CV diseases, and based on the meta-analysis, the pooled OR for CV diseases was 1.22 (95% CI 1.17–1.28) in the self-reported SHS individuals compared with the non-exposed group [36]. Investigators reported that SHS exposure caused structural and functional alterations on arterial walls including endothelial dysfunction and increased arterial stiffness leading to higher blood pressure. SHS results in the progression of atherosclerosis and a higher risk of coronary heart disease or stroke [37]. In addition, SHS contributes to respiratory infections and the onset or deterioration of asthma and has adverse effects on male and especially female fertility and, during pregnancy, low birth weight, head circumference and reduced fetal growth [16,25,38,39]. Considering this, patient 1’s prematurity could have been related to SHS exposure during pregnancy.

SHS exposure is associated with adverse effects on the baby’s lung growth and development. Babies exposed to SHS are more susceptible to respiratory diseases, asthma, and sudden infant death syndrome [38,39,40]. Young children are most affected by SHS and least able to avoid it. A meta-analysis even suggested that SHS exposure in children was a risk factor for attention deficit hyperactivity disorder and adverse behavioural outcomes [41,42]. Most children’s exposure to SHS comes from adults (parents or others) smoking at home [43]. For instance, 90% of children sharing a house with a smoker have detectable total nicotine and total cotinine levels in urine [44]. Epidemiological studies have suggested that exposure to SHS in childhood increases the risk of lung cancer later in life. In a case–control study (N = 2932), all individuals exposed to SHS had a higher risk of lung cancer (OR 1.30, 95% CI 1.08–1.7); however, the subjects first exposed before age 25 had a significantly higher lung cancer risk compared with those for whom first exposure occurred after the age of 25 years [45]. Similarly, the OR for subjects with exposure to SHS as a child and as an adult was 1.63 (95% CI 0.8–3.5) and for those only exposed as an adult 1.2 (95% CI 0.5–3.0) in another case-control study (N = 832) [46]. A significant impact of SHS in childhood was also detected when considering various histological types of lung cancer [31]. Both women from the presented cases were also exposed to SHS in their childhood.

Exposure to passive smoking occurs most often in homes and at work. The aforementioned pooled analysis of 18 case–control studies observed no association between SHS and lung cancer for individuals exposed only at work (OR 1.02, 95% CI 0.93–1.13), but positive associations were observed for those exposed at home (OR 1.19, 95% CI 1.08–1.31) and those exposed both at home and work (OR 1.39, 95% CI 1.27–1.52). The risk of lung cancer increased with increasing years of exposure at home (*p* < 0.001), at work (*p* = 0.02), and at home and work combined (*p* = 0.002) [31]. Ten years of work exposure was noted in case 2, but both patients were mainly exposed at home in childhood.

Public bans are an important non-price deterrent from smoking (firsthand and figuratively also SHS), ranked ahead of information campaigns, advertising bans, package warnings, and treatment for smokers. Legislative smoking bans have been shown to reduce youth smoking behaviour in public and reduce passive exposure in public places. But people tend to smoke more at home or in a car when smoking in public areas is banned [5,47,48]. This fact was shown in the Canadian cross-sectional health survey data of the population aged 12 and over, where in the period under review (2003–2012) bans on smoking in public were introduced across the provinces and territories. There was a 34% increase in people smoking in households [48]. On the other hand, as mentioned above, the prevalence of SHS in households has decreased in previous decades [17]. A literature review assessing the effects of various types of tobacco control legislation on smoking behaviour in developed countries showed that full bans reduced smoking prevalence over time, especially among younger demographic groups, but had no significant impact on the intensity of smoking among smokers. Partial bans did not significantly impact the prevalence of smoking [49]. Worldwide, tobacco-use prevention measures remain focused mainly on adolescents and young people [50]. Targeted efforts among the socioeconomically disadvantaged groups are needed too [22].

Smoke-free policies tend to be aimed primarily at enclosed public or workplace settings with very few countries attempting to control exposure in private or semi-private spaces such as homes and cars, and, as a result, children may be benefiting less from smoke-free measures than adults [51]. Households face challenges in setting no-smoking rules or are exposed to SHS drifting in from neighbouring homes. They often deal with agreeing on smoking bans and end up compromising on less effective strategies such as restricting smoking to specific parts of the home or smoking out of a window [52]. In particular, non-smoking households in multiunit housing may not be sufficiently protected from SHS by smoking bans [53].

Regarding smoke-free policies, there are considerable global differences. The most recent WHO Report on the Global Tobacco Epidemic (2021) indicates that 67 countries are classified as having complete smoke-free policies (defined as all public places completely smoke-free or at least 90% of the population covered by complete subnational legislation), 29 with moderate restrictions covering 6–7 types of public places, 43 with minimal policies in a small number of settings, and some 56 countries with either weak or no smoke-free policies or not reporting any data [54]. Compliance with the legislation also varies by country, and there is a need for education and empowerment together with guidance and changing social norms to help deliver the full benefits that smoke-free spaces can bring [51,55]. The 2019 WHO report provides details of local assessors rating overall compliance across various types of public spaces (on a score of 0–10) and indicated only 24 countries achieved high levels of compliance at 9–10 on the scale. In contrast, low levels of compliance (rating 0–3) were identified in 32 countries [51].

To explore the causal effect of SHS in lung carcinogenesis, exposure assessments should estimate chronic exposure to SHS on an individual basis. However, conventional exposure assessment for SHS relies on one-off or short-term measurements of SHS indices. In many studies, exposure estimates for cancer risk assessment are based on interview or questionnaire data and may therefore be debatable and open to criticism, especially when reporting long-term exposure. A more reliable approach would be to use biological markers that are specific for SHS exposure, e.g., cotinine in urine. This approach requires an understanding of the underlying mechanisms through which SHS could contribute to lung carcinogenesis [30]. As indicated in an original study (N = 1933) comparing self-reported smoking status with cotinine measurements, the questionnaire-based status may lead to an underestimation of smoking prevalence [56].

The possibility to compare and generalize conclusions of studies dealing with the effect of SHS is limited by the complexity of inhalation exposure with many variables influencing the effect. Moreover, some studies on SHS are based on current non-smokers, whereas others focus on never-smokers. But the definitions of a never-smoker are not unified: few studies define never smokers as those who have never smoked in their life. Many define never smokers as those who have smoked fewer than 100–400 (depending on the study) cigarettes [57].

## 5. Conclusions

At least a third of the world’s population is exposed to SHS. SHS harms our health especially if it occurs in childhood. Epidemiological studies demonstrate a causal relationship between SHS and lung cancer. Therefore, measures should be taken to reduce the prevalence of exposed children. When taking a personal smoking history, health care workers should focus not only on current or past firsthand smoking but also on current and past exposure to SHS for considering outcomes arising from SHS exposure in their patients. Data on SHS exposure should be considered for routine incorporation into electronic health records. Finally, the association of rarer histological types of lung cancer with SHS should be further studied.

## Data Availability

Not applicable.
